# Application of Modified Shell Vial Culture Procedure for Arbovirus Detection

**DOI:** 10.1371/journal.pone.0001034

**Published:** 2007-10-17

**Authors:** Edna R. Caceda, Tadeusz J. Kochel

**Affiliations:** American Embassy, Naval Medical Research Center Detachment, Lima, Peru; U.S. Naval Medical Research Center Detachment/Centers for Disease Control, United States of America

## Abstract

The isolation of arboviruses from patient's low titer sera can be difficult. Here we compared the detection efficiency of Dengue (DEN), Yellow Fever (YF), Saint Louis Encephalitis (SLE), West Nile (WN), Ilheus (ILH), Group C (GC), Oropouche (ORO), Mayaro (MAY) and Venezuela Encephalitis Equine (VEE) viruses using a Modified Shell Vial Culture (MSVC) protocol to a Standard Cell Culture (SCC) protocol. First the MSVC and SCC protocols were compared using five dilutions for each of the following stock viruses: DEN-1, DEN-2, DEN-3, DEN-4, YF, SLE, WN, ILH, GC, ORO, MAY and VEE. Next, patients' original sera from which viruses (DEN-1, DEN-2, DEN-3, YF, GC, ORO, MAY and VEE) had been previously isolated were compare by the two methods using five sera dilutions. In addition, seven sera that were positive for DEN-3 by RT-PCR and negative by SCC were processed by MSVC. The MSVC protocol was consistently 1-2 logs higher virus dilution more sensitive for virus detection than the SCC protocol for all stock *Flaviviruses* tested (DEN-1, DEN-2, DEN-3, DEN-4, YF, SLE, WN and ILH). MSVC was equal to or one log more sensitive for virus detection than SCC for the stock *Bunyaviruses* (GC and ORO). For the stock *Alphavirus* MAY, MSVC was equally or one log more sensitive for virus detection than SCC, while for VEE SCC was equally or one log more sensitive for virus detection than MSVC. MSVC was consistently one to two sera dilutions more sensitive than SCC for the detection of *Flaviviruses* from patients' sera. Both methods were approximately equally sensitive for the detection of *Bunyaviruses* from patients' sera and equal or one dilution less sensitive for the detection of *Alphaviruses* from patients' sera. Additionally, MSVC detected DEN virus in five of seven DEN-3 RT-PCR positive, SCC negative patients' sera.

## Introduction

The isolation of arboviruses from patient's low titer sera can be difficult.

Shell vial culture centrifugation methods for virus isolation have been shown to be more sensitive than standard isolation methods for members of the *Paramyxoviradae* [1]–[4], *Herpesviridae* [5], [6], *Orthomyxoviridae* [7] and *Flaviviridae* [8] families. DEN-2, Japanese Encephalitis (JE) and WN are the *Flaviviridae* viruses for which the Shell Vial method has been tested [9]. The original Shell Vial method has been adapted to 24 well tissue culture plates with centrifugation (Modified Shell Vial Culture (MSVC))[8].

We have compared the MSVC and standard cell culture (SCC) methods of virus detection for the *Flaviviruses*: DEN-1, DEN-2, DEN-3, DEN-4, YF, SLE, ILH and WN; the *Bunyaviruses*: GC, ORO and the *Alphaviruses*: MAY and VEE. The comparison was made using two cell lines, C636 and Vero, two incubation times, four and 10 days, with stock viruses and original patients' sera from which viruses had previously been isolated.

## Methods

### Stock viruses

DEN-1, DEN-2, DEN-3, DEN-4, YF, SLE, WN, ILH, GC, ORO, MAY and VEE viruses were propagated in Vero cells with Earle's Modified Essential Medium with 100 units/ml Penicillin, 100 ug/ml Streptomycin, 1 mM Sodium Pyruvate and 2% (V/V) Fetal Bovine Serum at 37°C. Upon observation of cytopathic effects, medias were collected, clarified by centrifugation at 40,000 g for five minutes and stored at −80°C until use. The *Flaviviruses* and *Bunyaviruses* were titered in BHK-21 cells; the *Alphaviruses* were titered in Vero cells.

### Original sera

Sera were collected from febrile subjects and processed for virus isolation following the SCC method (1∶5 inoculum, 10 day incubation, C636 and/or Vero culture) and stored at −80°C until use.

### The SCC method

100 ul of each stock virus dilution (undiluted, 100, 10, 1 and 0.1 PFU) or serum dilution (1∶5, 1∶10, 1∶50, 1∶100 and 1∶500) were inoculated in C636 and Vero cells propagated in T-25 cm^2^ flasks. The cells were incubated at 28°C and 37°C for C636 and Vero cells, respectively, for 4 and 10 days. The cells were scraped off the flasks, transferred to 5 ml test tubes and collected by centrifugation at 450 g for 10 minutes. The cell pellets were reconstituted with PBS and spotted onto slides and tested by indirect immunofluorescent assay (IFA) using DEN, YF, SLE, WN, ILH, GC, ORO, MAY and VEE polyclonal antibodies.

### The MSVC method

100 ul of each stock virus dilution (undiluted, 100, 10, 1 and 0.1 PFU) or serum dilution (1∶5, 1∶10, 1∶50, 1∶100 and 1∶500) were inoculated in C636 and Vero cells propagated in 24 wells plates. The plates were centrifuged at 680 g for 30 minutes and incubated at 33°C and 37°C for C636 and Vero cells, respectively, for 4 and 10 days. The cells were scraped off the plates, transferred to 5 ml test tubes and collected by centrifugation at 450 g for 10 minutes. The cell pellets were reconstituted with PBS and spotted onto slides and tested by IFA using DEN, YF, SLE, WN, ILH, GC, ORO, MAY and VEE polyclonal antibodies.

## Results and Discussion

For all stock *Flaviviruses* tested by the two methods, the MSVC method consistently required one to two logs less virus for virus detection in C636 cells than the SCC method (Table 1). The required amount of virus for detection by MSVC in Vero cells ranged from two logs less, to equal to that, required by the SCC method. For the *Bunyaviruses*, in both cell lines, the MSVC method required equal, or one log less, virus than SCC for virus detection. The results are mixed for the *Alphaviruses*. In both cell lines, MAY like the *Bunyaviruses*, the MSVC method required equal, or one log less, virus than SCC for virus detection. With VEE, MSVC required equal, or one log more, virus for virus detection than the SCC method (Table 1).

**Table 1:** Comparison of stock virus detection by SCC and MSVC.

VIRUS	Genera	C636 Cells Lowest concentration virus detected (PFU)				Vero Cells Lowest concentration virus detected (PFU)			
		Day 4 SCC	Day 4 MSVC	Day 10 SCC	Day 10 MSVC	Day 4 SCC	Day 4 MSVC	Day 10 SCC	Day 10 MSVC
DEN 1 (16007)	Flavivirus	100	1	10	0.1	10	1	0.1	0.1
DEN 2 (16681)	Flavivirus	4.7x10^4^	10	100	0.1	4.7×10^4^	100	4.7×10^4^	0.1
DEN 3 (IQD 1728)	Flavivirus	100	0.1	10	0.1	3.0×10^3^	100	100	1
DEN 4 (1036)	Flavivirus	10	0.1	10	0.1	100	10	1	0.1
YF (17D)	Flavivirus	3.2×10^5^	1	10	0.1	10	1	1	0.1
SLE (CDC)	Flavivirus	1	0.1	1	0.1	0.1	0.1	0.1	0.1
WNV (CDC)	Flavivirus	10	0.1	0.1	0.1	0.1	0.1	0.1	0.1
ILHEUS (ATCC)	Flavivirus	10	0.1	100	0.1	1	0.1	0.1	0.1
ILHEUS (FSE 0800)	Flavivirus	100	0.1	10	0.1	0.1	1	0.1	0.1
GROUP C (IQU 1719)	Bunyavirus	1	0.1	0.1	0.1	0.1	0.1	0.1	0.1
OROPOUCHE 172	Bunyavirus	10	1	10	0.1	10	1	1	1
MAYARO (TRVL15537)	Alphavirus	10	1	10	1	10	1	10	10
VEE (TC 83)	Alphavirus	0.1	1	0.1	0.1	0.1	0.1	0.1	1

The two methods were compared for virus detection from sera from which virus had been isolated following the SCC method. For the *Flavivirus* sera (table 2, sera 1–18) MSVC consistently required one to two dilutions less sera for virus detection than SCC. Additionally, the MSVC method identified virus in four sera that were negative for virus detection by SCC after four days of incubation (table 2: Vero cells serum 7; C636 cells sera 13,15 and 17). Virus could not be re-isolated from three sera (table 2, sera 10–12) by SCC while virus was detected after only four days of incubation by MSVC. For the *Bunyaviruses*, in both cell lines, the MSVC method required equal, or one dilution less, serum for virus detection than SCC (table 2, sera 19–27). For the *Alphavirus* sera, MSVC required more serum for virus detected after four days of incubation for three of seven specimens in C636 culture (table 2, sera 28, 30 and 31) than SCC. However, after ten days of incubation the two methods required equal amounts of sera for virus detection for six of the seven sera. One serum (# 34) was negative for virus detection by MSVC but positive by SCC, in both cell lines.

**Table 2:** Comparison of virus detection in sera by SCC and MSVC.

Serum #	SCC (C6/36 1∶5 Isolate)	RT-PCR	C636 Cells Highest dilution virus detected				Vero Cells Highest dilution virus detected			
			Day 4 SCC	Day 4 MSVC	Day 10 SCC	Day 10 MSVC	Day 4 SCC	Day 4 MSVC	Day 10 SCC	Day 10 MSVC
1	D1		1∶500	1∶500	1∶500	1∶500	1∶100	1∶500	1∶500	1∶500
2	D1		1∶500	1∶500	1∶500	1∶500	1∶50	1∶100	1∶500	1∶500
3	D1		1∶500	1∶500	1∶500	1∶500	1∶100	1∶500	1∶500	1∶500
4	D2		1∶50	1∶100	1∶100	1∶500	NEG	1∶10	1∶5	1∶10
5	D2		1∶100	1∶500	1∶500	1∶500	1∶5	1∶10	1∶10	1∶10
6	D3		1∶500	1∶500	1∶500	1∶500	1∶500	1∶500	1∶500	1∶500
7	D3		1∶100	1∶500	1∶100	1∶500	NEG	1∶500	1∶500	1∶500
8	D3		1∶500	1∶500	1∶500	1∶500	1∶500	1∶500	1∶500	1∶500
9	D3		1∶5	1∶500	1∶5	1∶500	NEG	NEG	NEG	NEG
10	D3		NEG	1∶100	NEG	1∶500	NEG	NEG	NEG	NEG
11	D3		NEG	1∶100	NEG	1∶500	NEG	NEG	NEG	NEG
12	D3		NEG	1∶5	NEG	1∶500	NEG	NEG	NEG	NEG
13	D3		NEG	1∶500	1∶5	1∶500	NEG	NEG	NEG	NEG
14	D3		1∶50	1∶500	1∶50	1∶500	NEG	NEG	NEG	1∶10
15	D3		NEG	1∶500	1∶10	1∶500	NEG	NEG	NEG	NEG
16	D3		1∶100	1∶500	1∶500	1∶500	NEG	NEG	1∶500	1∶500
17	D3		NEG	1∶10	1∶50	1∶500	NEG	NEG	NEG	NEG
18	YF		1∶100	1∶500	1∶100	1∶500	1∶50	1∶500	1∶100	1∶500
19	GC		1∶50	1∶500	1∶100	1∶500	1∶5	1∶10	1∶5	1∶10
20	GC		1∶100	1∶500	1∶500	1∶500	1∶10	1∶50	1∶100	1∶100
21	GC		1∶50	1∶500	1∶100	1∶500	1∶50	1∶500	1∶50	1∶500
22	ORO		1∶500	1∶500	1∶500	1∶500	1∶500	1∶500	1∶500	1∶500
23	ORO		1∶500	1∶500	1∶500	1∶500	1∶500	1∶500	1∶500	1∶500
24	ORO		1∶500	1∶500	1∶500	1∶500	1∶500	1∶500	1∶500	1∶500
25	ORO		1∶500	1∶500	1∶500	1∶500	1∶500	1∶500	1∶500	1∶500
26	ORO		1∶500	1∶500	1∶500	1∶500	1∶500	1∶500	1∶500	1∶500
27	ORO		1∶500	1∶500	1∶500	1∶500	1∶500	1∶500	1∶500	1∶500
28	MAY		1∶500	1∶100	1∶500	1∶500	1∶5	1∶5	1∶10	1∶50
29	MAY		1∶500	1∶500	1∶500	1∶500	1∶10	1∶10	1∶10	1∶50
30	MAY		1∶500	1∶100	1∶500	1∶500	1∶500	1∶100	1∶500	1∶500
31	VEE		1∶500	1∶100	1∶500	1∶500	1∶500	1∶500	1∶500	1∶500
32	VEE		1∶500	1∶500	1∶500	1∶500	1∶500	1∶500	1∶500	1∶500
33	VEE		1∶500	1∶500	1∶500	1∶500	1∶500	1∶500	1∶500	1∶500
34	VEE		1∶50	NEG	1∶5	NEG	1∶50	NEG	1∶10	NEG
35	NEG	D3	NEG	NEG	1∶5	1∶50	NEG	NEG	NEG	NEG
36	NEG	D3	NEG	NEG	NEG	NEG	NEG	NEG	NEG	NEG
37	NEG	D3	NEG	NEG	NEG	NEG	NEG	NEG	NEG	NEG
38	NEG	D3	NEG	1∶10	NEG	1∶50	NEG	NEG	NEG	NEG
39	NEG	D3	NEG	1∶10	NEG	1∶50	NEG	NEG	NEG	1∶500
40	NEG	D3	NEG	1∶50	NEG	1∶5	NEG	1∶50	1∶10	1∶500
41	NEG	D3	1∶10	1∶500	1∶100	1∶500	NEG	NEG	NEG	1∶500

To further compare the two methods, seven SCC negative, DEN-3 RT-PCR positive sera were assayed by MSVC. MSVC identified virus in five of seven sera after 10 days of incubation in C636 culture while SCC detected virus in three of the sera (table 2, sera 35–41).

The SCC method maybe less reproducible for virus detection than MSVC. DEN virus could not be detected in three sera (table 2, sera 10, 11 and 12) from which the viruses were originally isolated by SCC and three SCC negative, DEN-3 RT-PCR positive sera were SCC positive for DEN virus (table 2, sera 35, 40 and 41). Possibly those sera have low DEN virus titers that are at or near the limit of virus detection by SCC. However, all six specimens were positive for DEN virus by MSVC.

MSVC is a rapid and efficient method for the isolation of *Flaviviruses* (DEN-1, DEN-2, DEN-3, DEN-4, YF, SLE, WN and ILH), *Bunyaviruses* (ORO and GC) and the *Alphavirus* MAY. The SCC method could be more suitable for the isolation of the *Alphavirus* VEE. For studies that involve the isolation of arboviruses the utilization of both C636 MSVC and Vero SCC may maximize virus isolation.
